# Supplementary material for: Assessment of genomic prediction capabilities of transcriptome data in a barley multi-parent RIL population
Source: Theor Appl Genet. 2025 Sep 10;138(10):247. doi: 10.1007/s00122-025-05029-0 (PMC12423136; doi:10.1007/s00122-025-05029-0)
Supplement: Supplementary file 1 — (pdf 3372 KB) [file 122_2025_5029_MOESM1_ESM.pdf]

SUPPLEMENTARY MATERIAL

Assessment of genomic prediction capabilities of transcriptome data in a barley multi-parent RIL population

Table S1. GP performance changes of all dataset combinations compared to SNP_{Array}^{Total} (here: 1.4). All traits with significantly increased prediction abilities (Up), significantly decreased prediction abilities (Down), and not significantly changed prediction abilities (NC) were counted and named (EL: ear length, AL: awn length, PH: plant height, FT: flowering time, GL: grain length, GW: grain width, GA: grain area, TGW: grain weight). Dataset names are analogous to Fig. 5.

Dataset	Down	Up	NC	Decreased traits	Increased traits
1.1	7	1	0	EL,PH,FT,GL,GW,GA,TGW	AL
1.2	2	5	1	GA,TGW	EL,AL,FT,GL,GW
1.3	7	0	1	AL,PH,FT,GL,GW,GA,TGW	
1.4	0	0	8		
2.1	2	4	2	GA,TGW	EL,AL,FT,GL
2.2	7	1	0	EL,PH,FT,GL,GW,GA,TGW	AL
2.3	1	4	3	GA	EL,AL,FT,GW
2.4	4	4	0	PH,GW,GA,TGW	EL,AL,FT,GL
2.5	1	6	1	GA	EL,AL,FT,GL,GW,TGW
2.6	3	3	2	PH,GA,TGW	EL,AL,FT
3.1	4	4	0	PH,GW,GA,TGW	EL,AL,FT,GL
3.2	1	5	2	GA	EL,AL,FT,GL,GW
3.3	3	3	2	PH,GA,TGW	EL,AL,FT
3.4	1	5	2	GA	EL,AL,FT,GL,GW
4.1	2	5	1	GA,TGW	EL,AL,FT,GL,GW

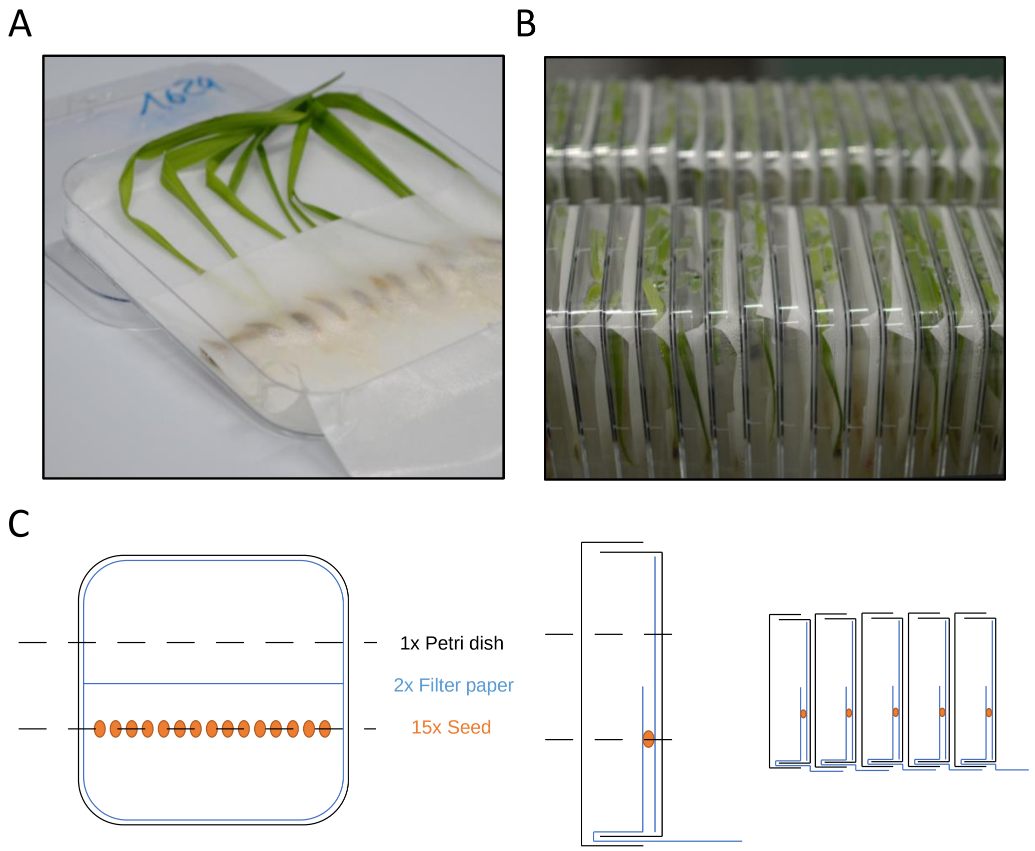

Fig. S1. Illustration of the cultivation system of (A) barley seedlings in Petri dishes using (B) space efficient cultivation. (C) Schematic overview of the approach used to cultivate barley seedlings in reach-in growth chambers.

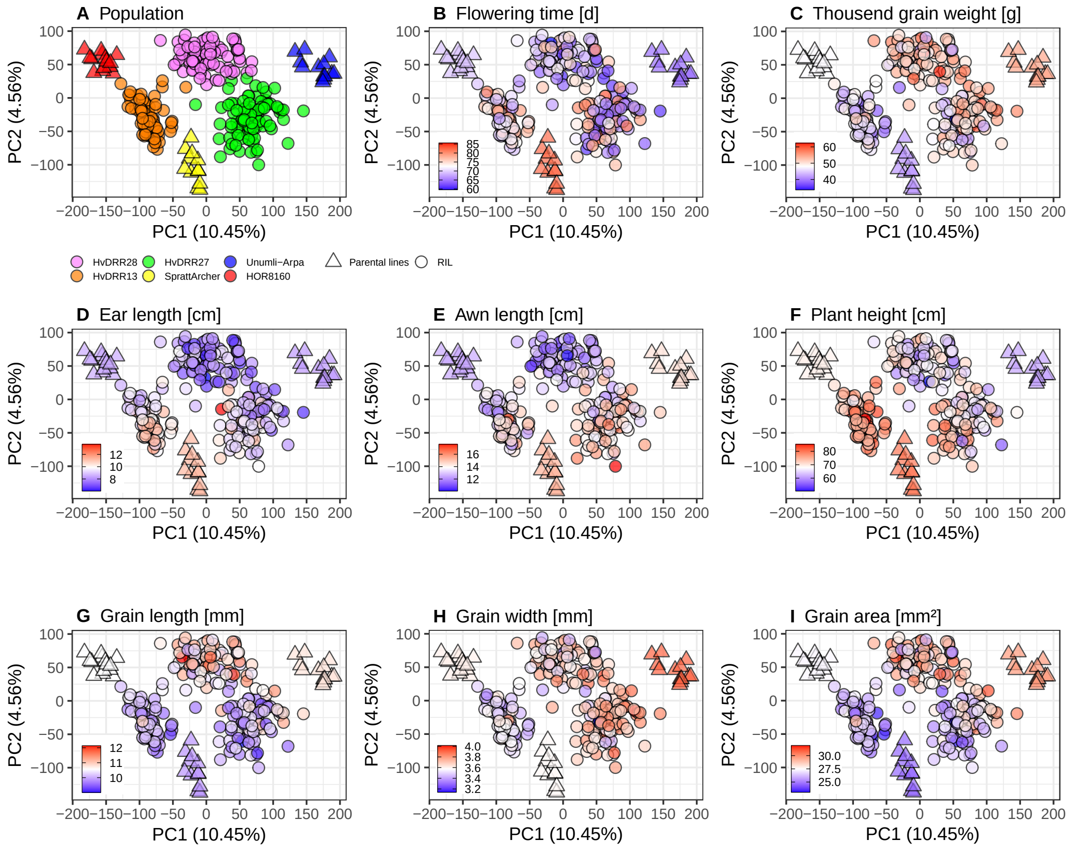

Fig. S2. Principal component analysis (PCA) based on GE^{DEG}_{RNAseq}, (A) overlayed with the information of the genetic material and (B-I) the adjusted entry means of all eight traits. Shape differentiate RILs and parental inbreds. PC 1 and PC 2 are the first and second principal component, respectively, and the number in parentheses refers to the proportion of variance explained by the principal components in percent.

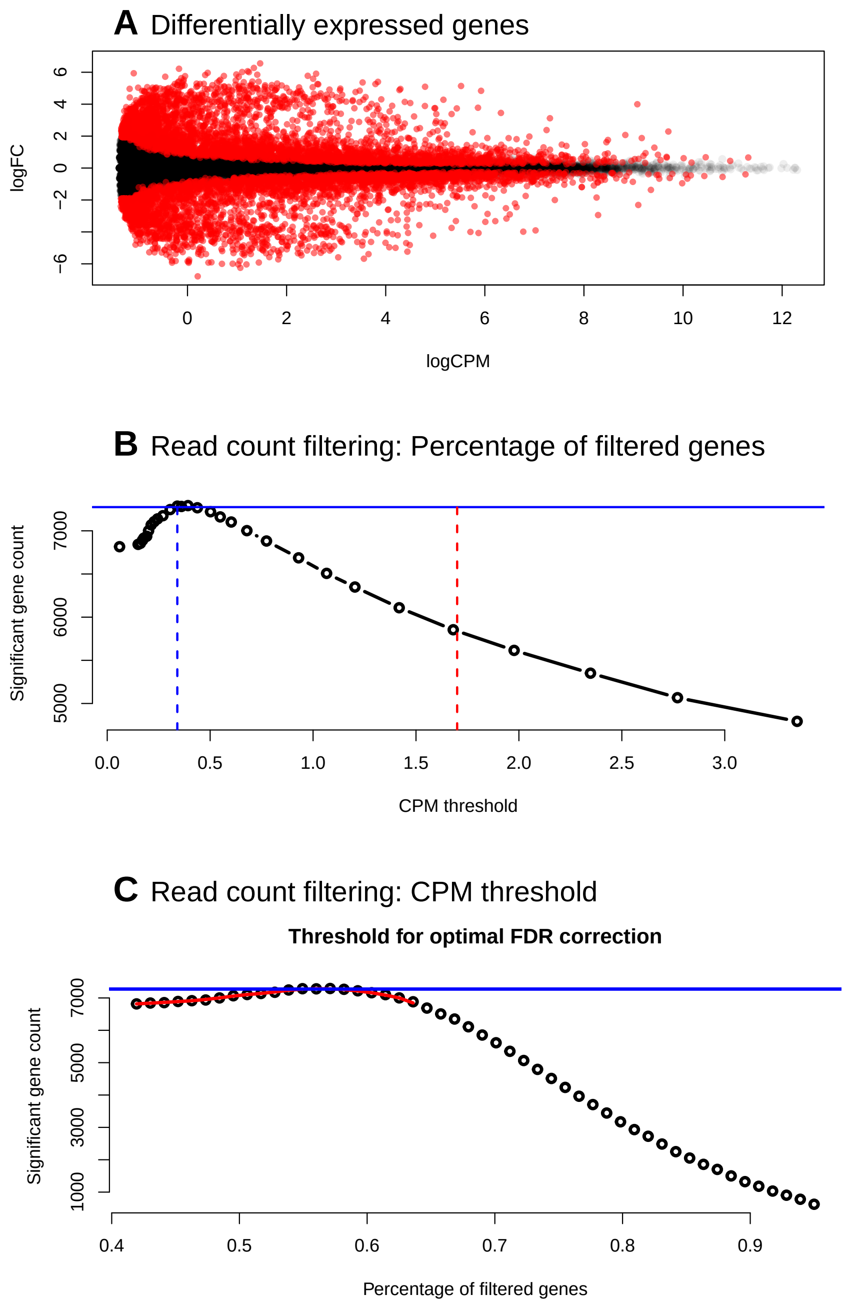

Fig. S3. overview of the read count filtering. (a) the number of differentially expressed genes shown in red could be maximized by adjusting the percentage of (b) filtered transcripts or (c) cpm threshold. the blue horizontal line indicating the maximum of differentially expressed genes counted. the dotted vertical lines show the chosen threshold (blue) and the edger standard threshold (red).

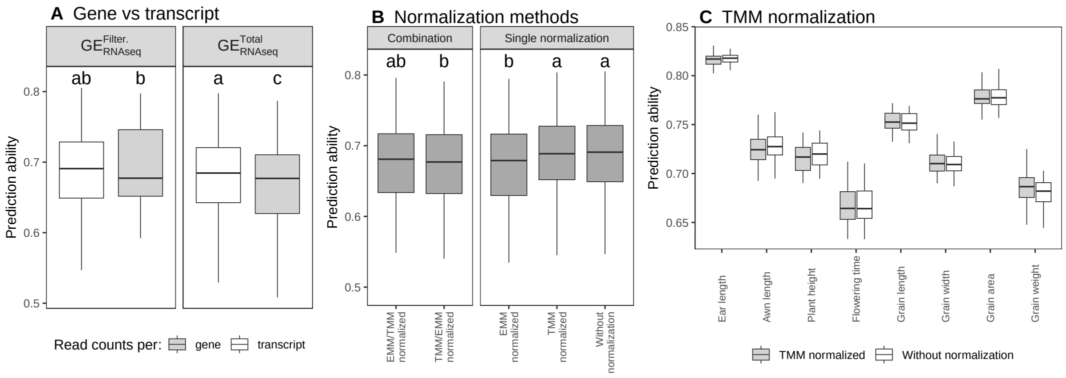

Fig. S4. Characterization of the expression data. (A) Comparison of the genomic prediction results for two different read counting methods: counting per gene and counting per transcript for GE^{Total}_{RNAseq} and $GE^{Filter.}_{RNAseq}$. (B) Genomic prediction results for GE^{DEG}_{RNAseq} compared based on the order of normalization: Trimmed Mean of the M-values (TMM) first or estimated marginal means (EMM) based on block effect first. (C) Genomic prediction abilities for GE^{DEG}_{RNAseq} comparing unnormalized and TMM normalized data for all eight traits.

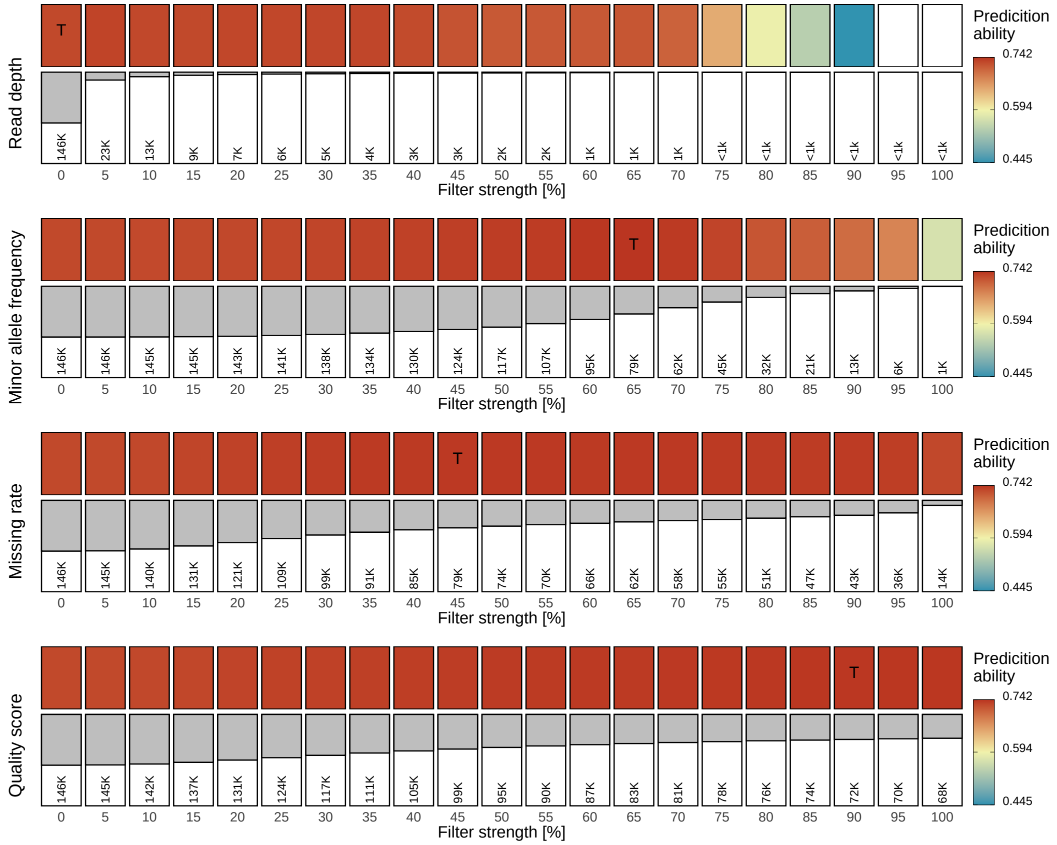

Fig. S5. Quality filtering of genomic variants of sequencing data based on genomic prediction (GP) performance. The performance shown are prediction abilities averaged across all eight traits. For each of the four criteria the prediction ability (top) and the remaining number of markers in thousands (bottom) are shown for 21 different relative filtering strength subsets from the minimum (0%) to the maximum (100%) value in the original dataset (SNP^{Total}_{RNAseq}). The best-performing filter strength subset is marked (T). No GP was performed (white) when the number of remaining markers was insufficient.

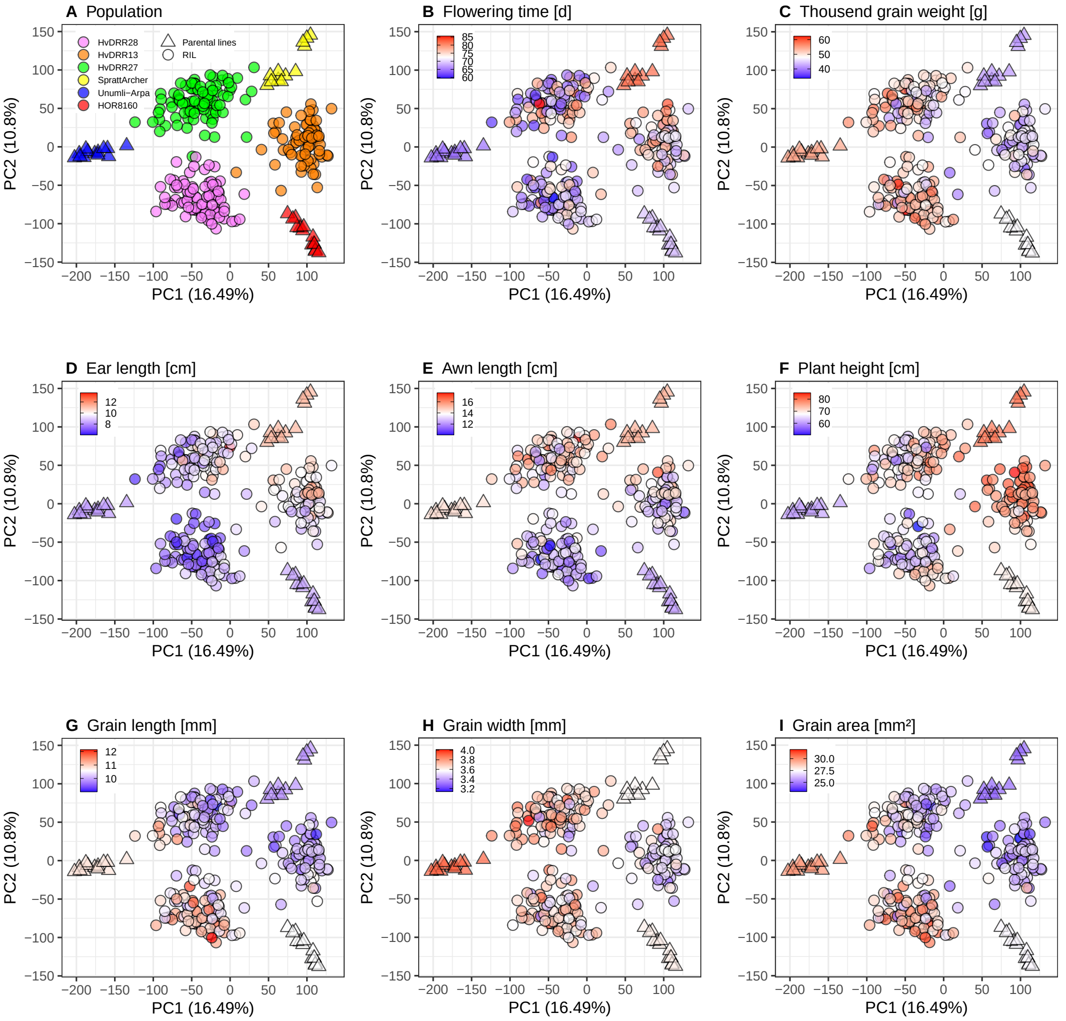

Fig. S6. Principal component analysis (PCA) based on SNP^{QC}_{RNAseq}, (A) overlayed with the information of the genetic material and (B-I) the adjusted entry means of all eight traits. Shape differentiate RILs and parental inbreds. PC 1 and PC 2 are the first and second principal component, respectively, and the number in parentheses refers to the proportion of variance explained by the principal components in percent.

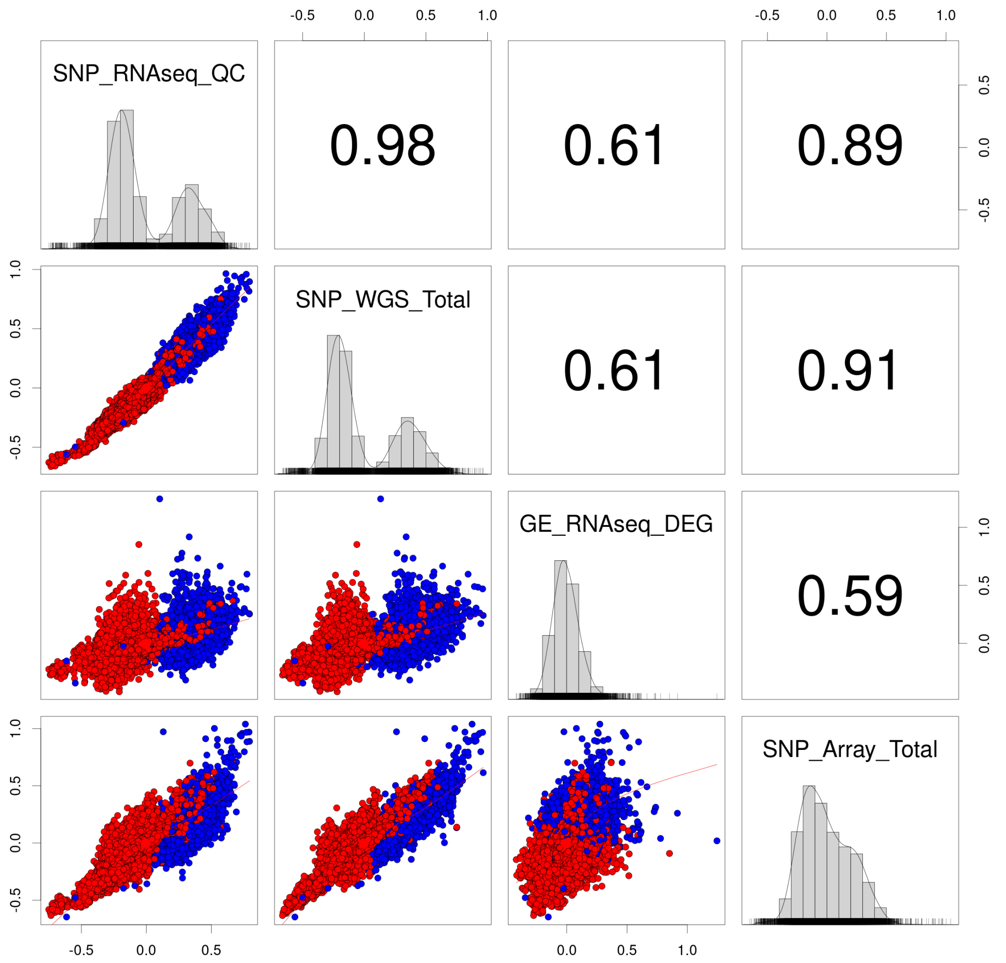

Fig. S7. Correlation plots of the additive relationship matrices of the datasets: SNP^{QC}_{RNAseq} (SNP_RNAseq_QC), SNP^{Total}_{WGS} (SNP_WGS_Total), GE^{DEG}_{RNAseq} (GE_RNAseq_DEG), SNP^{Total}_{Array} (SNP_Array_Total). The dots are colored by sample relationship, with intra-population comparisons blue and inter-population comparisons red.

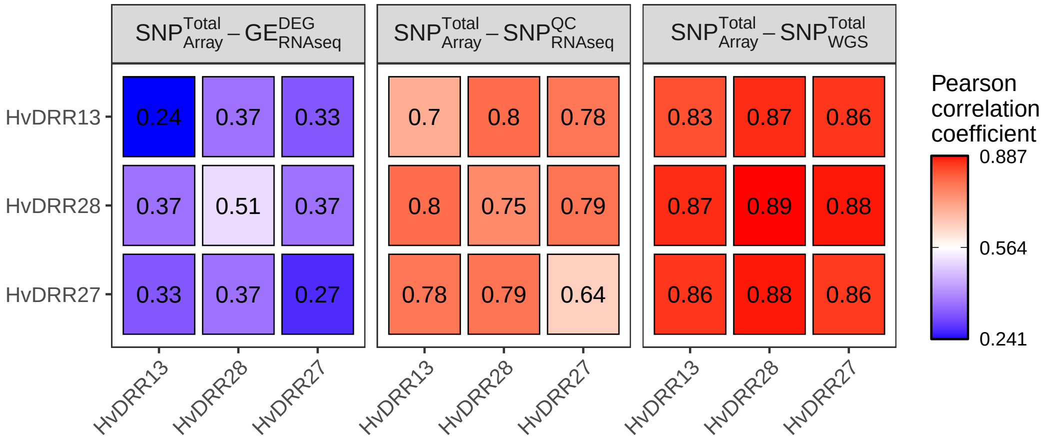

Fig. S8. Intra and Inter-population additive relationship matrices comparison. Heat map of Pearson correlation coefficients between the covariances of population segments of two additive relationship matrices. Comparison between SNP^{Total}_{Array} and the three remaining main datasets: SNP^{QC}_{RNAseq}, SNP^{Total}_{WGS}, and GE^{DEG}_{RNAseq}.

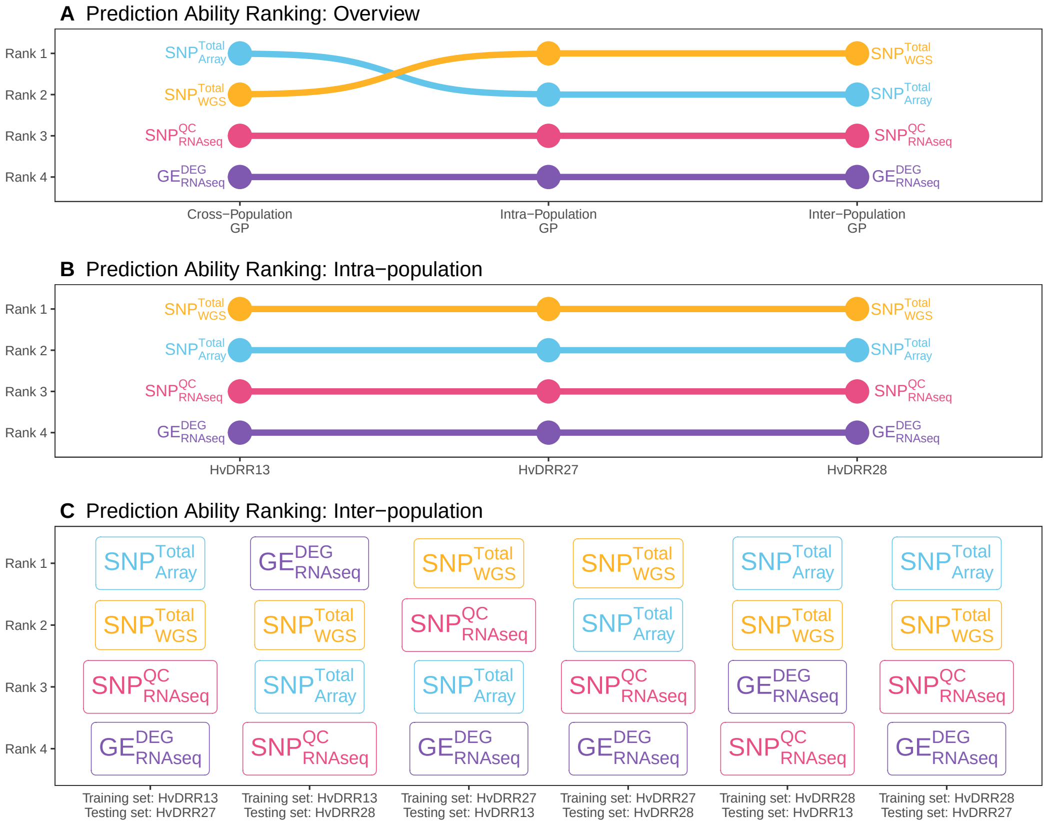

Fig. S9. Prediction Ability ranking for (A) intra-population and (B) inter-population comparisons using the best-performing datasets from each data origin group. The training set size was set to 50 for all tests.

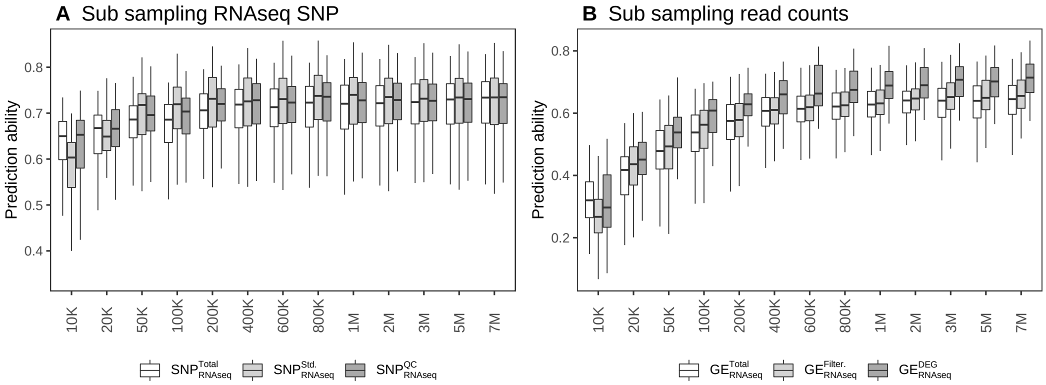

Fig. S10. Genomic prediction (GP) performance of artificially reduced sequencing depth subsets created by random read sub-sampling of (A) RNA-Seq genomic variant datasets (SNP^{Total}_{RNAseq}, SNP^{QC}_{RNAseq}) and (B) gene expression datasets (GE^{Total}_{RNAseq}, $GE^{Filter.}_{RNAseq}$, GE^{DEG}_{RNAseq}) including 155 of the 240 samples. The sub-sampling ranged in 13 steps between 10 thousand to 7 million reads.

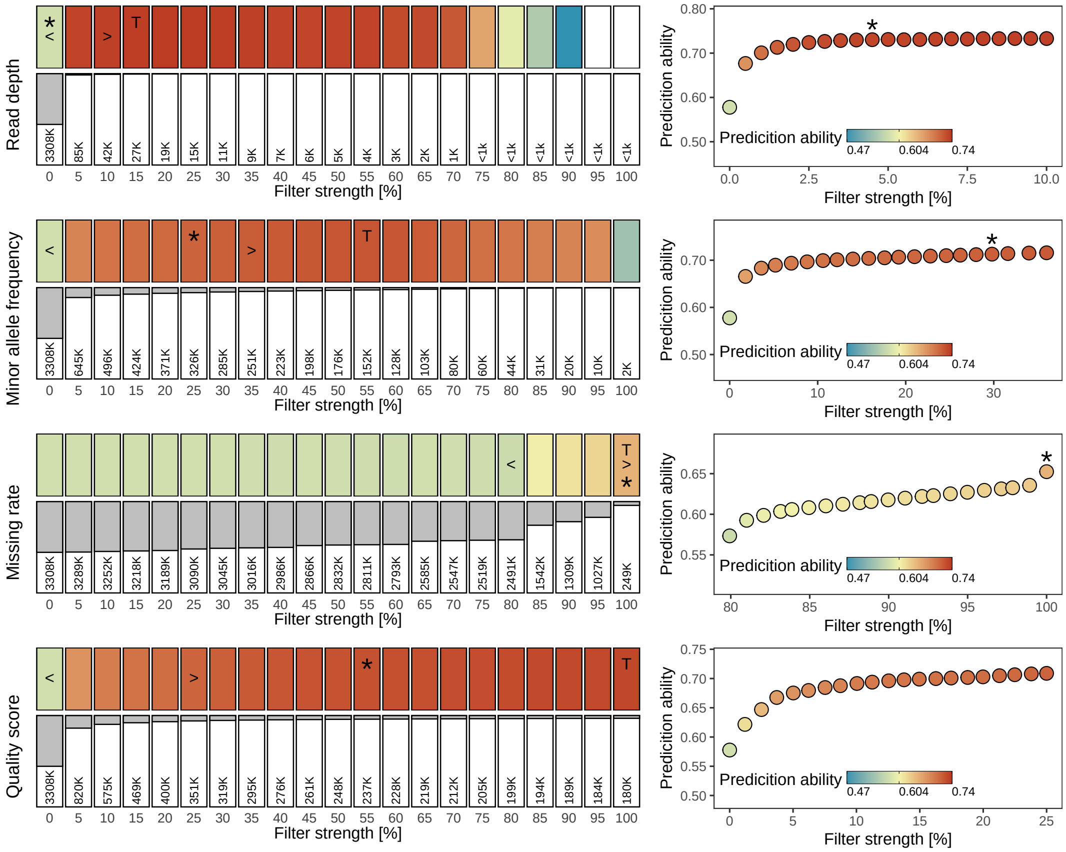

Fig. S11. Quality filtering of genomic variants of sequencing data based on genomic prediction (GP) performance. Quality filtering is an essential step in maximizing the GP potential and can have a larger effect on the results. We tested the impact of quality filtering on a less strictly cleaned dataset by creating SNP^{Raw}_{RNAseq}, an alternative version of SNP^{Total}_{RNAseq}, which included heterozygous / inconsistent allele calls and marker with missing parental data. From the 5.9M raw variants SNP^{Raw}_{RNAseq} included 3.4M, which is much more then SNP^{Total}_{RNAseq} (148K) and it resulted in a reduction GP performance by 0.15, but applying the same quality filtering workflow resulted in a subset with a comparable GP performance to SNP^{QC}_{RNAseq}.

The performance shown are prediction abilities averaged across all eight traits. For each of the four criteria the prediction ability (left, top) and the remaining marker in thousands (left, bottom) are shown for 21 different relative filtering strength subsets from the minimum (0%) to the maximum (100%) value in the original dataset (SNPTotal RNAseq). The best-performing filter strength subset is marked top (T) as well as the optimal filter strength subset (*). The filtering subset marked * are not significantly ($p > 0.05$) different to T. The filter strength region with the highest impact on GP performance is shown in detail (right). The section is outlined on the overview as from start (<) to end (>).
